# Pharmacological Properties of *Allium cepa*, Preclinical and Clinical Evidences; A Review

**DOI:** 10.22037/ijpr.2020.112781.13946

**Published:** 2021

**Authors:** Farzaneh Kianian, Narges Marefati, Marzie Boskabady, Seyyedeh Zahra Ghasemi, Mohammad Hosein Boskabady

**Affiliations:** a *Department of Physiology, School of Medicine, Tehran University of Medical Sciences, Tehran, Iran. *; b *Department of Physiology and Medical Physics, Faculty of Medicine, Baqiyatallah University of Medical Sciences, Tehran, Iran. *; c *Dental Materials Research Center and Department of Pediatric Dentistry, School of Dentistry, Mashhad University of Medical Sciences, Mashhad, Iran. *; d *Applied Biomedical Research Center, Mashhad University of Medical Sciences, Mashhad, Iran. *; e *Department of Physiology, School of Medicine, Mashhad University of Medical Sciences, Mashhad, Iran. *; 1 *F. K. and N. M. contributed equally to this work*

**Keywords:** Onion, Allium cepa, Quercetin, Thiosulphinates, Phenolic acids, Pharmacological properties

## Abstract

Onion or *Allium cepa* (*A. cepa*) is one of the most important condiment plants grown and consumed all over the world. This plant has various therapeutic effects attributed to its constituents, such as quercetin, thiosulphinates and phenolic acids. In the present article, various pharmacological and therapeutic effects of *A. cepa* were reviewed. Different online databases using keywords such as onion, *A. cepa*, therapeutic effects, and pharmacological effects until the end of December 2019 were searched for this purpose. Onion has been suggested to be effective in treating a broad range of disorders, including asthma, inflammatory disorders, dysentery, wounds, scars, keloids and pain. In addition, different studies have demonstrated that onion possesses numerous pharmacological properties, including anti-cancer, anti-diabetic and anti-platelet properties as well as the effect on bone, cardiovascular, gastrointestinal, nervous, respiratory, and urogenital systems effects such as anti-osteoporosis, anti-hypertensive, antispasmodic, anti-diarrheal, neuro-protective, anti-asthmatic and diuretic effects. The present review provides detailed the various pharmacological properties of onion and its constituents and possible underlying mechanisms. The results of multiple studies suggested the therapeutic effect of onion on a wide range of disorders.

## Introduction

*Allium cepa* (*A. cepa*) is a bulbous perennial or biennial monocot plant belonging to the family *Liliaceae* and the genus *Allium*, which includes over 250 genera and 3700 species (1-4). It is believed that the origin of *A. cepa* is central Asia, possibly the regions of Iran and Pakistan. However, it has now been distributed all over the world, such as Europe, North America and Africa (2, 5). *A. cepa* has several vernacular names like Onion in English, Cyvannulli in Malayalam, Pyaj in Hindi, Miruli in Kannada and Piaz in Farsi. The flowers of onion are small and usually purple or white. The root system of this plant is superficial and the stem at its base is very short flattened which increases in diameter as growth continues. The leaves of onion are long, linear, hollow and cylindrical. When the plant reaches a certain stage of growth, the leaf bases become thick and form a bulb (5). Onion is categorized based on their color into yellow, red and white and based on their taste as sweet and non-sweet (6, 7). This plant is used in many forms, including raw bulbs, fresh juice, fried and roasted (8-11).

Onion has been known that possesses various medicinal properties from ancient times (12). The plant is traditionally used to treat different diseases such as cough due to bronchitis, asthma, inflammatory disorders, dysentery, ulcer wounds, scars, keloids, pain and swelling after bee or wasp stings (13). Experimental researches have also shown numerous pharmacological effects for onion, including decreases blood levels of cholesterol, triglycerides and thromboxanes (substances involved in the development of cardiovascular disease), inhibits platelet aggregation and platelet-mediated thrombosis (a process resulting in heart attacks and strokes), functions as a hypoglycemic, neuroprotective, anti-convulsant, anti-hypertensive, anti-depressant and diuretic agent, protective effect on the liver, prevents the processes of oxidation and inflammation as well as the release of histamine associated with asthma, stimulates the immune system and decreases osteoporosis (14-32). Moreover, onion is one of the strongest anti-carcinogenic components because it suppresses the growth of carcinogenic cells (33).

This article reviews the pharmacological properties of onion and its constituents along with possible mechanisms in various fields.

##  Experimental 

*Materials and Methods*

Databases including Google Scholar, PubMed, Medline, ISI Web of Knowledge, Science Direct and Scopus were searched for the terms such as “onion”, “*Allium cepa*”, “flavonoid”, “organosulfur”, “phenolic compounds”, “clinical trial” and “animal studies” to identify studies on pharmacological effects of *A. cepa* and constituents of onion and possible underlying mechanisms of these effects.

## Results

*Chemical constituents*

Numerous effects of onion may be due to a wide range of constituents, including quercetin, thiosulphinates and phenolic acids which have often been reported from this plant. The preliminary phytochemical investigations have showed that onion contains water, carbohydrates (*e.g.* inulin, fructooligosaccharides, isorhamnetin-4-glucoside, galactose, glucose and mannose), proteins, vegetal hormone (glycoquinine), lectin, steroids, essential oils (*e.g. *catechol protocatechnic acid, thiocyanate and thiopropiono aldehyde), phytoestrogens (*e.g. *coumestrol, zearalenol, isoflavones and humulone), vitamins (*e.g.* A, B complex, C and E), minerals (*e.g.* selenium, phosphorus, iron, calcium and chromium), flavonoids (*e.g. *quercetin, apigenin, rutin, myricetin, kaempferol, catechin, resveratrol, epigallocatechol-3-gallate, luteolin and genistein), organosulfuric compounds (*e.g.* thiosulphinates, cepaenes, cysteine, S-methyl cysteine sulfoxide, diallyl disulfide, allyl methyl sulfide, allyl propyl disulfide, gamma-L-glutamyl-trans-S-1-propenyl-L-cysteine sulfoxide, S-propenyl cysteine sulfoxide, S-alk(en)yl cysteine sulfoxides and S-allyl cysteine sulfoxide) and allicin (*e.g. *Diallyl disulfide, diallyl trisulfide and ajoene), phenolic compounds (*e.g. *phenolics, phenolic acids, anthocyanins and hydroxycinnamic acid), lipophilic antioxidants (*e.g. *dialkyl disulfides), aglycones, anthocyanin, saponins and fistulosin (octadecyl 3-hydroxyindole) (5, 8, 27 and 34-48).

There are different flavonoids in onion skin, including quercetin aglycone, quercetin diglucoside, quercetin 4-glucoside and isorhamnetin monoglycoside or kaempferol monoglycoside (49). More than 80 percent of flavonoids exist in the outer scales of the onion because of exposure to sunlight. The plant also contains 1–5 percent dry weight of S-alk(en)yl cysteine sulfoxides that are precursors of its aroma (50).

*Anti-cancer effects*

Compelling evidence indicates that individuals who consume copious amounts of onion, their susceptibility to cancer at various organ sites is reduced (51-53). Polyphenolics such as flavonoids found in onion are partially responsible for this beneficial effect (42, 54). In addition, organosulfur compounds (*e.g.* cysteine, S-methyl cysteine, diallyl disulfide and diallyl trisulfide) content of onion play an important role in cancer chemoprevention as several studies are showing protective effects of these compounds against liver, stomach, colorectal and breast cancers (55-60). Moreover, onion, due to high-selenium content, can prevent some cancer occurrence without resulting in an excessive accumulation of tissue selenium, a concern associated with standard selenium compounds (61, 62).

Several mechanisms have been proposed to explain the cancer-preventive effects of onion and related constituents. For example, quercetin is able to prevent reactive oxygen species (ROS)-induced DNA damage and mutational changes, inhibit the activity of tyrosine kinases which are oncogenic and increase the bioavailability of some anti-cancer drugs (*e.g.* tamoxifen) by promoting their intestinal absorption and decreasing their metabolism (63-65). In the case of organosulfur compounds, it is suggested that they induce phase II detoxifying enzymes, including quinone reductase and glutathione S-transferase and increase the activities of glutathione reductase, glutathione peroxidase (GPx) and superoxide dismutase (SOD) leading to carcinogen detoxification and elimination (66-68). These compounds also affect sulfhydryl/disulfide exchange reactions or induce apoptosis that are probably crucial for controlling cell proliferation and tumor growth (69-71). Moreover, organosulfur compounds stimulate glutathione (GSH) synthesis, the enzyme necessary for conjugating GSH to electrophiles and blocking DNA-adduct formation (72). Other mechanisms that organosulfur compounds exert anti-cancer effects include modulation of multidrug resistance enzymes which are very useful for improving the response of cancers to chemo-therapies, blockade of the formation of the cancer-causing substance, inactivation of cancer-causing substances and an increase of DNA repair (73-75).

*In-vitro studies*

Some studies have demonstrated anti-cancer effects of onion on different malignant cells *in-vitro*. For example, the preventive effect of onion oil on skin tumor promotion through an increase in GSH activity was reported (72). In the study done by Wang *et al.*, ethyl acetate extract of onion inhibited fatty acid synthase and induced apoptosis in fatty acid synthase over-expressing human breast cancer cells. Since obesity is closely associated with breast cancer, the results of this study suggested that onion might be valuable for preventing obesity-related malignancy (53). The anti-proliferative effect of onion on gastric and breast cancers in human cells by inducing apoptosis was also demonstrated (76). In addition, Shrivastava *et al.* investigated the protective effects of aqueous extract of onion on Melanoma cells. This study revealed significant activity of onion as a cytoprotective agent of normal cells and cytotoxicity agent for tumor cells as a considerable decrease in Melanoma cell population by crude extract of the plant (77). Another *in-vitro *study evaluated the effects of onion against telomerase activity of HL-60 cells and exhibited both telomerase inhibitory effect and cancer cell proliferation inhibitory effect with onion administration (78). 

There is also substantial *in-vitro *research that has examined the anti-cancer effects of different compounds of onion. For example, one study indicated anti-cancer effects of polyphenols in human leukemia cells by inducing caspase-dependent apoptosis. The apoptosis was triggered through the extrinsic pathway by up-regulating death receptor 5 as well as by the intrinsic pathway by a down-regulating cellular inhibitor of apoptosis 1 family member (79). Another study approved the potency of quercetin against human breast cancer by inhibiting cancer cell growth and induction of apoptosis in cancer cells (80). Several studies on quercetin, an important ingredient of onion, has shown that this agent could be an anticarcinogen for numerous cancer types including breast cancer, leukemia, colon, squamous cell, ovary, endometrial, gastric and non-small-cell lung (81-90). Quercetin was also found to down regulate the expression of mutant *p53* protein in human breast cancer cell lines resulting in arresting cells in the G2-M phase of the cell cycle (91, 92).

Moreover, the investigators reported the anti-proliferative effects of diallyl disulfide and diallyl trisulfide on cultured human neoplastic and non-neoplastic lung cells (93). In 2002, it was shown a significant dose-dependent cytotoxic response of diallyl disulfide on ascites tumor cells (94). It has also been demonstrated that treatment of human tumor cells with diallyl disulfide resulted in the complete cessation of growth through a proportional increase in the percentage of cells arrested in the G2-M phase of the cell cycle (95, 96).

*In-vivo studies*

There are epidemiological and experimental studies that indicate the anti-cancer effects of onion. In a French epidemiological study, a higher intake of onion was associated with a lower risk of breast cancer (97). Another study done in northeast China revealed an inverse relationship between onion consumption and brain cancer (98). Also, decreased risk of gastric cancer with increased intake of onion has been reported (99). In case-control investigations, it was reported that onion consumers had a lower risk of large bowel cancer compared to controls (51). The relationship between onion intake and incidence of colorectal adenoma was also assessed using 562 cases and 5,932 controls from subjects in the prostate, lung, colorectal, and ovarian cancer screening trial which demonstrated the protective effects of onion intake on these cancers. In another study, Chen *et al.* indicated that Taiwanese onion consumers had a lower risk of esophageal cancer (100, 101). These beneficial effects of onion consumption have also been observed in oral cavity/pharyngeal, laryngeal, lung, pancreatic, prostate and ovarian cancers (88, 102-104). Moreover, an experimental study was designed to evaluate the protective effect of onion on colorectal cancer with hyperlipidemia. This study showed that the proliferation of colorectal cancer with onion administration was significantly inhibited in mice (105). 

**Figure 1:**
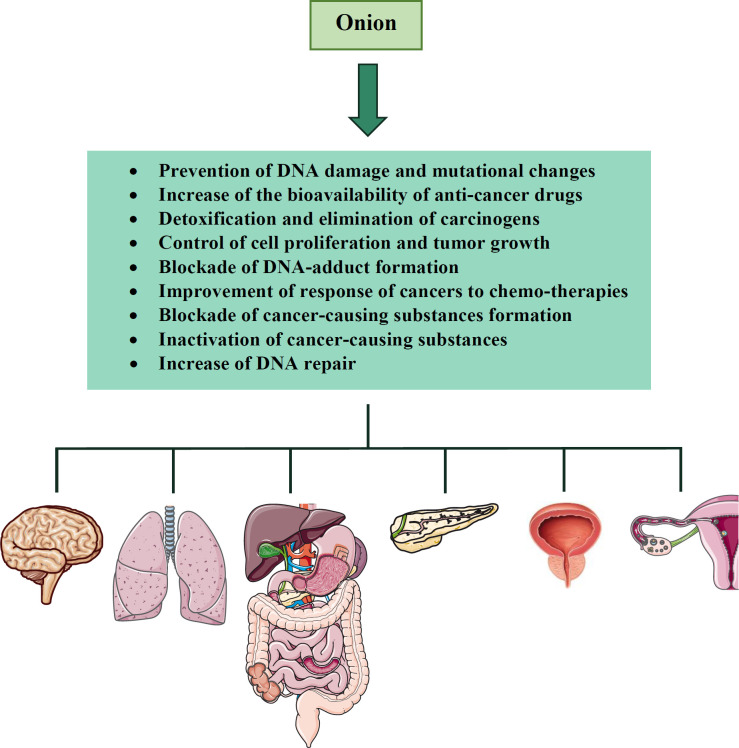
The anti-cancer effects of *A. cepa* (onion) and its constituents on different organs

Several pieces of evidence also demonstrate the anti-cancer effects of different constituents found in onion. Former studies have reported that high intakes of flavonoids are correlated with a lower risk of cancer (102). In patients with advanced cancers, intravenous administration of quercetin inhibited the proliferation of cancerous cells (106). Similarly, in the study of Molnar *et al.*, quercetin when was given intraperitoneally to animals with ascetic tumors showed anti-tumor effects (107). Another animal study examined the effect of quercetin on mice bearing abdominal tumors derived from a human pharyngeal squamous cell carcinoma line. The result of this study revealed that quercetin exerted significant inhibition of tumor growth (86). Besides, the protective effect of organosulfur compounds (*e.g.* diallyl disulfide and diallyl trisulfide) against carcinogenesis was examined in animal models. When administered to mice, those constituents inhibited pulmonary adenoma formation, gastric neoplasia and growth of hepatoma (108, 109). Moreover, the anti-tumor properties of diallyl disulfide were shown in various experimental studies (110, 111). Another compound found in onion, steroidal saponins also have anti-cancer potential against lung carcinoma, melanoma and leukemia (112, 113). Anti-cancer effects of onion and its constituents were shown in Table 1. The summary of the anti-cancer effects of onion was demonstrated in Figure 1.

According to the studies mentioned above, onion acts as an anti-cancer agent by increasing detoxification enzymes, preventing cell division and inhibiting cellular proliferation. Some constituents found in onion also have anti-cancer properties. For example, quercetin prevents mutational changes, inhibits the activity of tyrosine kinases and increases the bioavailability of some anti-cancer drugs. Allins and quercetin are able to inhibit the growth of malignant cells. Organosulfur compounds have chemo-preventive activity.

*Anti-diabetic effects*

The available evidence shows that onion possesses anti-diabetic effects (114-118). One of the most important applications of onion in traditional medicine is its use for the treatment of diabetes to lower blood glucose levels. Extracts of onion also modulate the liver hexokinase activity glucose 6-phosphatase and HMG coenzyme-A reductase and normalize the plasma level of lipid and glucose. Consuming onion results in hypoglycemia in diabetic patients (1). Treatment of streptozotocin (STZ)-induced diabetic rats with red onion extract showed decreased fasting blood glucose (FBG) levels and improved glycation end products (AGEs). Increased serum insulin levels and suppressing inflammatory mRNA expression were also observed in treated animals with the extract which were due to hyperglycemia (119). In another study, 100 g daily intake of onion in both type1 and 2 diabetic patients resulted in a significant decrease in FBG compared to insulin which was about 89 and 40 mg/dL in type 1 and 2 diabetic patients, respectively.

**Figure 2:**
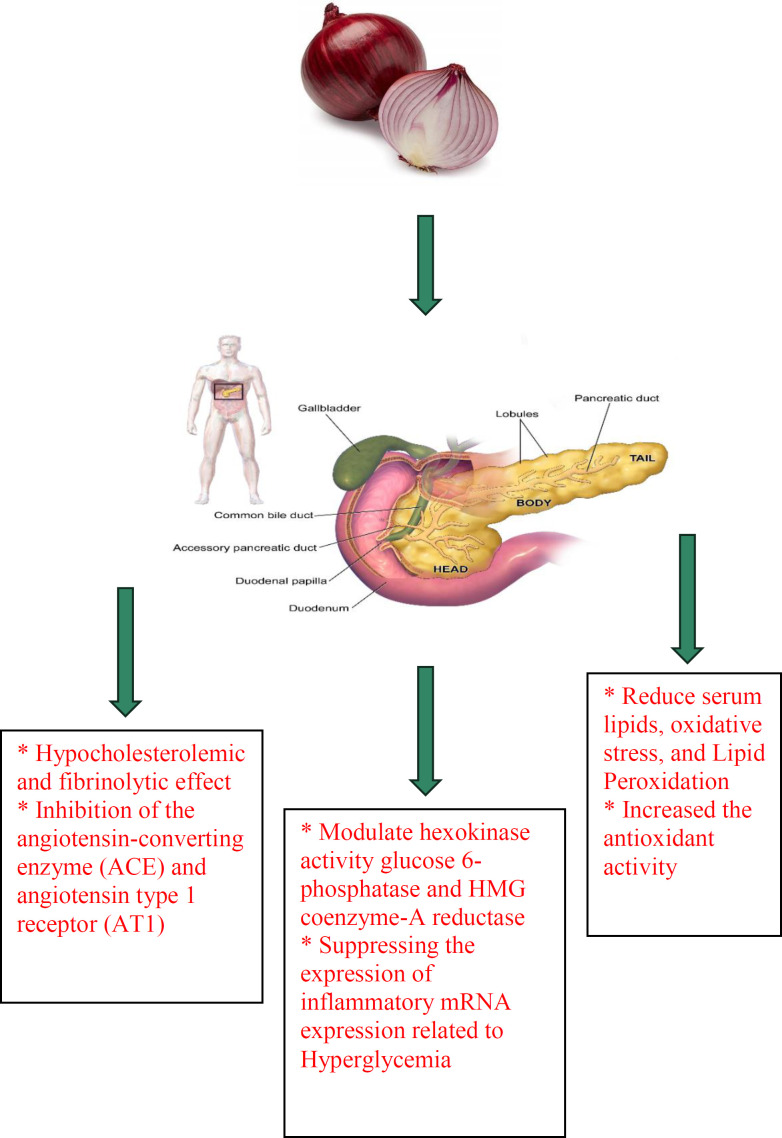
The anti-diabetic effects of *A. cepa* (onion) and its constituents. ACE: angiotensin-converting enzyme, AT1: angiotensin type 1 receptor

The results suggest that the anti-diabetic effect of onion could be due to the recovery or reproduction of pancreatic beta cells and improves the activity of enzymes in the pathways of glycolysis and gluconeogenesis (120). Oral supplementation of onion in STZ-induced diabetic rats resulted in decreasing nitric oxide production in diabetic animals. Therefore, orally onion treatment increased the expression of cardiac inducible nitric oxide synthase (iNOS) protein and mRNA expression resulting in reduced cardiac vascular damage due to diabetes (121). Since diabetes is associated with oxidative stress due to the compromise of natural antioxidant mechanisms and an increase in oxygen-free radical production, the reduction of oxidative stress may have potential therapeutic effects (122). Various studies have demonstrated that treatment with onion causes a decrease in free radicals because of the presence of components such as S-methyl cysteine sulfoxide (123, 124). This component also stimulates insulin secretion, controls blood glucose and normalizes the activities of liver hexokinase, glucose 6-phosphatase and HMG-CoA reductase enzymes (123, 125). S-allyl cysteine sulfoxide, another constituent found in onion, has been reported to have therapeutic effects on diabetes by stimulating the production and secretion of insulin, interfering with dietary glucose absorption and favoring insulin saving (126, 127). In addition, allyl propyl disulfide and chromium content in onion have ability to decrease fasting blood levels of glucose and insulin and increase glucose tolerance (5). Quercetin by inhibiting α-glucosidase is also able to prevent the release of D-glucose from oligosaccharides and disaccharides resulting in delayed absorption of glucose from intestine and controlling blood glucose levels (128, 129). Additionally, quercetin can improve glucose-induced insulin secretion and protect pancreatic islets against oxidative damage through phosphorylation of extracellular signal-regulated kinase (ERK) (130, 131). Anti-diabetic effects of onion and its constituents were indicated in Table 2. The anti-diabetic effects of onion were demonstrated in Figure 2.

In summary, onion has anti-diabetic effects due to the presence of various constituents. For example, S-methyl cysteine sulfoxide can stimulate insulin secretion, control blood glucose and normalize the activities of liver hexokinase, glucose 6-phosphatase and HMG-CoA reductase enzymes. S-allyl cysteine sulfoxide stimulates the production and secretion of insulin, decreases dietary glucose absorption and saves insulin. Allyl propyl disulfide and chromium decrease fasting blood levels of glucose and insulin and increase glucose tolerance. Quercetin controls blood glucose levels, improves glucose-induced insulin secretion and protects pancreatic islets. 

*Anti-platelet effects*

**Figure 3:**
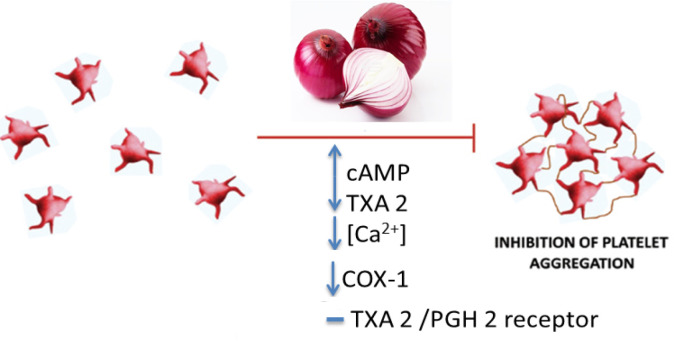
The anti-platelet effects of *A. cepa* (onion) and its constituents. cAMP: cyclic adenosine monophosphate, COX-1: cyclooxygenase-1, PGH2: prostaglandin H2, TXA2: thromboxane A2

The researchers have demonstrated that administration of onion essential oil increases the coagulation time and fibrinolytic activity (5). Onion has been reported to reduce platelet aggregation by blocking thromboxane and prostaglandin biosynthesis and preventing thrombus growth and promotion of fibrinolysis. This plant also increases the coagulation time and fibrinolytic activity (132-136). Concerning the mechanism of the anti-platelet effect of onions, it can be noted that onion peel and bulbs extract through its constituents, quercetin, adenosine, allicin, and paraffinic polysulphides with upregulation of cyclic adenosine monophosphate (cAMP) levels and down regulation of thromboxane A2 (TXA2) through reducing the [Ca2+], cyclooxygenase (COX)-1 and TXA2 synthase activities with TXA2/prostaglandin (PG) H2 receptor blockade lead to inhibition of platelet aggregation (17, 137). Also, onion extract reduces platelet aggregation without interfering with COX activity on arachidonic acid (138). Another mechanism for these properties was that sulfur, trisulfide, allicin, adenosine and quercetin were able to reduce TXB2 biosynthesis via suppression of COX-1 (139). Also, trisulfides which are the major constituent of onion through two mechanisms showed anti-platelet activity, including 1) inhibition of TXA2 biosynthesis by suppressing thromboxane synthase and 2) reduction of TXB2 biosynthesis by inhibiting COX-1 (140). It may appear that the mentioned properties of the plant are due to the presence of organosulfur components (35, 133 and 141). However, non-sulfur compounds present in onion such as quercetin and some phenolics can also act as anti-platelet agents (35, 142). Anti-platelet effects of onion and its constituents were shown in Table 2. The summary of the anti-platelet effects of onion was demonstrated in Figure 3.

*Bone effects*

**Figure 4.**
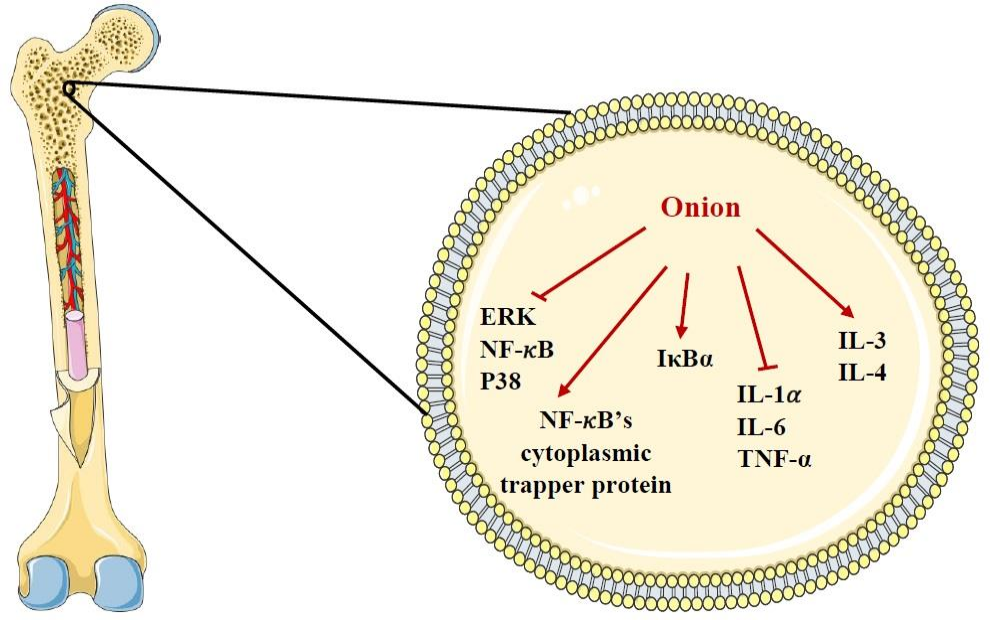
The effects of *A. cepa* (onion) and its constituents on bone cell. ERK: extracellular signal-regulated kinase IκBα: IkappaB-alpha, IL-1𝛼: interleukin-1𝛼, IL-3: interleukin-3, IL-4: interleukin-4, IL-6: interleukin-6, NF-𝜅B: nuclear factor kappa-light-chain-enhancer of activated B cells, TNF-𝛼: tumor necrosis factor-alpha

Onion has been shown to inhibit the activation and formation of osteoclast precursor cells and bone desorption as the plant decreases urinary excretion of [H3]-tetracycline, an index of bone desorption (143-146). Similarly, the study done by Wetli *et al.* indicated that one gram of onion added to the food of rats considerably inhibited bone reabsorption (147). In another experimental study, supplementing the diet of rats with onion powder also led to significant inhibition of bone reabsorption (148). It was demonstrated that onion administration attenuated the activation of ERK, p38 mitogen-activated protein kinases and nuclear factor kappa-light-chain-enhancer of activated B cells (NF-κB) induced by receptor activator of nuclear factor-kappa Β ligand (RANKL) (149). Because of the beneficial effects of onion on bones, it might be essentially valuable for menopause women who are at risk of osteoporosis (5). It has been suggested that one of the mechanisms of bone protection by onion is attributed to its base excess which is claimed to maintain calcium in bones (32, 150). In addition, quercetin present in onion has potential benefits for bone health by modulating inflammatory processes, downregulating NF-𝜅B, increasing NF-𝜅B’s cytoplasmic trapper protein expression and maintaining IkappaB-alpha (IκBα). Along with this, one *in-vitro *study showed that quercetin exerted an inhibitory effect on osteoclastic differentiation via a mechanism involving NF-κB and activator protein 1 (151). Quercetin also can inhibit the secretion of cytokines like interleukin (IL)-1𝛼, IL-6 and tumor necrosis factor-alpha (TNF-𝛼) which are involved in osteoclastogenesis and stimulate the secretion of IL-3 and IL-4 which are responsible for inhibition of osteoclast formation (152). Moreover, 1-propenyl-cysteine sulfoxide isolated from onion can inhibit osteoclast activity (5). Other constituents in onion such as rutin, several phytoestrogens (*e.g.* coumestrol, zearalenol, isoflavones and humulone) and vitamins (*e.g.* K and C) have been proposed to might be worthwhile for bone health (37, 46-48, 143 and 153). The effects of onion and its constituents on bone were shown in Table 3. The summary of the effects of onion on the bone cell was demonstrated in Figure 4.

Considering the above mentioned, onion has protective effects on bone via inhibiting the activation and formation of osteoclast precursor cells and bone desorption due to the presence of constituents such as quercetin, 1-propenyl-cysteine sulfoxide, phytoestrogens (*e.g.* coumestrol, zearalenol, isoflavones and humulone) and vitamins (*e.g.* K and C).

*Cardiovascular effects*

Methanol-soluble extract of onion on ischemic injury in heart-derived H9c2 cells *in-vitro *and in rat hearts *in-vivo *inhibited increases in the ROS, mitochondrial membrane depolarization, cytochrome c release and caspase-3 activation during hypoxia in H9c2 cells. In the *in-vivo *rat myocardial infarction model, onion extract considerably decreased the infarct size, apoptotic cell death of heart and plasma malondialdehyde (MDA) level. Therefore, the methanolic extract of onion showed a preventive effect on ischemia/hypoxia-induced apoptotic death in H9c2 cells and rat heart (154). In another study, it was found that the aqueous extract of onion could act as a cardioprotective agent against myocardial injury (155). A study indicates that aqueous onion extract reduces aortic nicotinamide adenine dinucleotide phosphate (NADPH) oxidase activity and lipid peroxidation resulting in reducing systolic blood pressure (156). Along with this, Nausheen *et al.* demonstrated that the antioxidant activity of onion leaves extract resulted in protection against doxorubicin-induced cardiotoxicity in rats (157). In another experimental study, the antihypertensive effects of onion in NG-nitro-L-arginine methyl ester-induced hypertensive rats due to the antioxidant activity of onion were shown (158). The plant may also be considered as a heart tonic agent (159). In addition, onion is able to induce endothelium-dependent vasorelaxation and increase vascular cyclic guanosine monophosphate (cGMP) levels (160).

**Figure 5.**
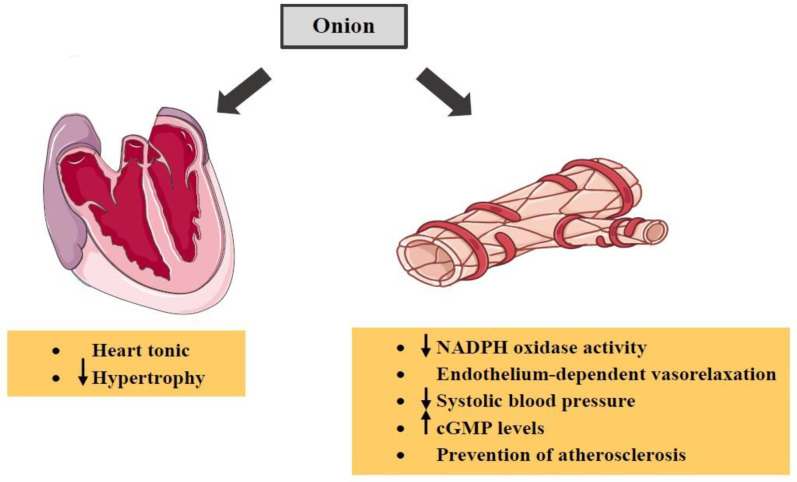
The effects of *A. cepa* (onion) and its constituents on cardiovascular system. ↓: Decrease; ↑: Increase; cGMP: cyclic guanosine monophosphate;NADPH: nicotinamide adenine dinucleotide phosphate

Moreover, the effect of onion extract on serum concentration of cholesterol, triglycerides and low-density lipoproteins (LDL) was examined and the results showed that serum lipids were significantly reduced consequent consumption of onion extract (161). Some evidence has shown that quercetin found in this plant protects against coronary heart disease (162-164). Quercetin also decreases elevated blood pressure and cardiac hypertrophy (165). A group of researchers have suggested that the effect of this constituent on suppressing the elevation of blood pressure may be through the increase in nitric oxide (NO) availability and nitric oxide synthase (NOS) activity and decrease in angiotensin II production (166). Furthermore, quercetin prevents atherosclerosis by inhibiting lipid peroxidation (167). S-alk(en)yl cysteine sulfoxide, another component of onion, has been shown to reduce serum LDL cholesterol, protect LDL cholesterol from oxidation and raise high-density lipoproteins (HDL) cholesterol (168-170). Moreover, some studies have demonstrated that S-methyl cysteine sulfoxide isolated from onion possesses anti-oxidative and hypolipidemia properties (171-173). The effects of onion and its constituents on cardiovascular were indicated in Table 3. The summary of various effects of onion on cardiovascular system was shown in Figure 5.

In summary, onion can be considered as a protective cardiovascular agent because it is able to reduce systolic blood pressure. Several constituents of this plant have also shown these beneficial effects. For example, quercetin causes decreases in elevated blood pressure, cardiac hypertrophy and atherosclerosis. S-alk(en)yl cysteine sulfoxide reduces serum LDL cholesterol and raises HDL cholesterol. In addition, S-methyl cysteine sulfoxide results in hypolipidemia.

*Gastrointestinal effects*

**Figure 6.**
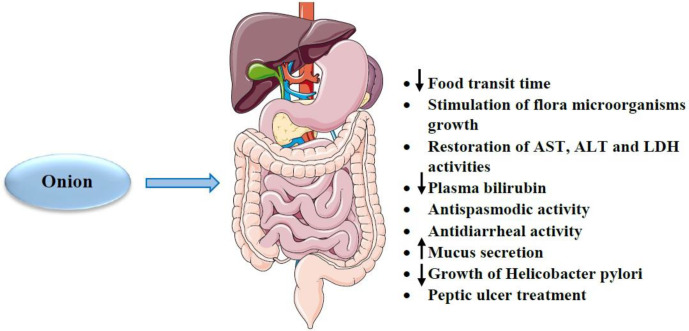
The effects of *A. cepa* (onion) and its constituents on gastrointestinal system. ↓: Decreas; ↑: Increase; ALT: alanine transaminase; AST: aspartate aminotransferase; LDH: lactate dehydrogenase

Onion has been indicated to reduce food transit time in the gastrointestinal tract (174). Some studies have also shown that this plant stimulates the growth of useful microorganisms such as bifidobacteria and lactobacilli in the colon due to its high soluble fiber content, including inulin and fructooligosaccharides (40, 175). Another study revealed that onion administration for 28 consecutive days restored the activities of aspartate aminotransferase (AST), alanine transaminase (ALT) and lactate dehydrogenase (LDH) enzymes to their normal levels and reduced plasma bilirubin (123). In addition, it has been reported that flavonoids (*e.g.* quercetin) content in onion has anti-spasmodic and anti-diarrheal activities (176). Moreover, quercetin appears to have a therapeutic potential in treating peptic ulcers by promoting mucus secretion and the inhibitory effect on Helicobacter pylori growth (177, 178). Administration of 1.5, 02 and 2.5 g/kg of onion powder to chicks led to increased weight gain and feed consumption. Also, supplementation of onion reduced the population of E. coli and enhanced Lactobacillus and Streptococcus species. In addition, this study showed that onion increased the morphometry of length, width, crypt depth and surface area of the villus in the duodenum, jejunum and ileum of the small intestine (179). Feeding with a combination of onion and coconut for 8 days to sheep with gastrointestinal nematodes and cestodes resulted in increased body weight and apparently worm reduction (180). Maitham *et al.* showed that onion bulb extract with doses of 100–200 mg/kg reduced the size of the dextran sulfate sodium-induced colitis. Also, this extract led to a decrease in pro-inflammatory cytokines and signaling pathways such as mitogen-activated protein kinase family, rapamycin, COX-2 and tissue inhibitors of metalloproteinases. In addition, the expression of interferon-γ (IFN-γ), C-X-C chemokines and factors involved in apoptosis such as cytochrome c, caspase-3 and 8 were reduced with onion bulb extract (181). The effects of onion and its constituents on gastrointestinal were summarized in Table 4. Various effects of onion on the gastrointestinal system were summarized in Figure 6.

According to the above mentioned, onion has protective effects on the gastrointestinal system. This plant causes the reduction of food transit time in the gastrointestinal tract and stimulation of useful microorganism growth, normalization of AST, ALT and LDH activities. Some onion constituents have also shown these beneficial properties. For example, flavonoids (*e.g.* quercetin) have anti-spasmodic and anti-diarrheal activities. In addition, quercetin promotes mucus secretion and inhibits Helicobacter pylori growth resulting in the mitigation of peptic ulcers.

*Nervous system effects*

**Figure 7.**
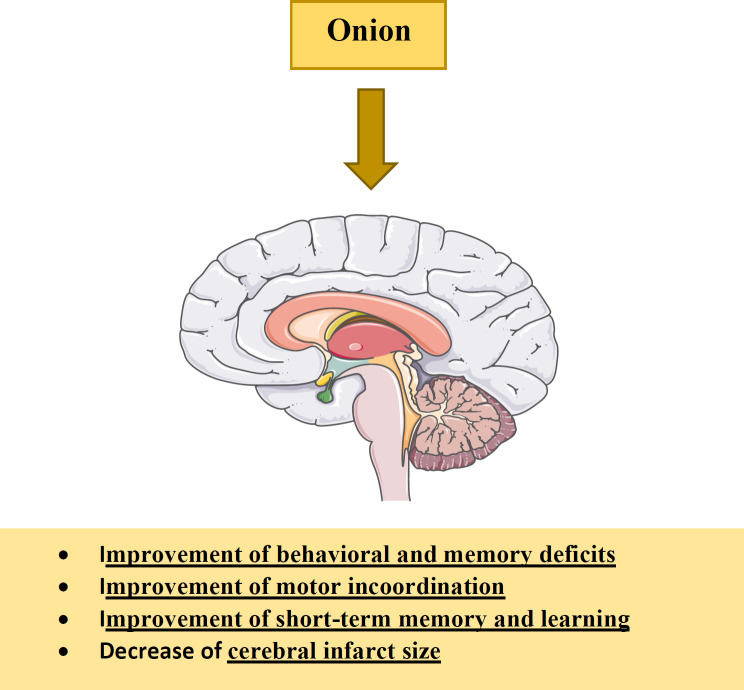
The effects of *A. cepa* (onion) and its constituents on nervous system

It has been shown that onion administration causes improvements in behavioral deficits, motor incoordination and short-term memory induced by cerebral ischemia-reperfusion (IR) through decreasing LDL cholesterol and lipid peroxidation while increasing GSH and catalase (CAT) activities (5, 182). The anti-oxidative effects of this plant are attributed to bioactive constituents such as flavonoids and organosulfur compounds (19, 183-186). The protective effects of methanolic extract of onion on cerebral IR were examined. The results indicated that administration of the extract reduced thiobarbituric acid reactive substances (TBARS) concentration in brain mitochondria and cerebral infarct size and prevented motor incoordination (19). Tamtaji *et al.* reported that onion ethanolic extract (125 and 250 mg/kg/day) for 4 weeks improves learning and memory performances, decreases the escape latency and traveled distance, and increases step-through latency in the Morris water maze and passive avoidance tests (187). Hydro-alcoholic extract of onion for 60 days improves motor incoordination and memory deficit, decreases oxidative stress and increases acetylcholinesterase (AChE) in neurotoxicity induced by aluminum chloride (182). Treatment with onion extract (200 mg/kg/day), dissolved in water for 14 days, also affects single immobilization stress-induced biochemical and behavioral changes in mice by decreases in the immobilization stress-induced anxiety and brain lipid peroxidation as well as increase of antioxidant enzymes activities. Therefore, these findings suggest that onion supplementation may be helpful in the treatment of anxiety and depression (188). The effects of onion and its constituents on the nervous system were shown in Table 4. Various effects of onion and its components on the nervous system were shown in Figure 7. 

As indicated, onion shows the protective effects on the nervous system through improvements in behavioral deficits, motor incoordination and short-term memory and reduction of cerebral infarct size.

*Respiratory effects*

The available data indicates that onion treatment can protect from bronchial asthma (189, 190). In an animal study, the aqueous extract of onion caused the reduction of lung inflammation and neutrophil, eosinophil and lymphocyte count (12). Another report showed that the methanolic onion extract decreased inflammatory cytokines (*e.g.* IL-5 and IL-13) and eosinophil peroxidase activity. The extract also relaxes tracheal smooth muscle (191). On the other hand, onion has been demonstrated to reduce vascular permeability resulting in a decrease of protein exudation in bronchoalveolar lavage fluid (29). Also, treatment of asthmatic rats with onion showed a significant decline in tracheal response to methacholine and decreased total WBC and phospholipases A2 (PLA2) levels compared to the untreated asthmatic group (192). In addition, the antioxidant and immunomodulatory properties of onion were demonstrated in sensitized rats by decreasing of nitrogen dioxide (NO2), nitrate (NO3), immunoglobulin E (IgE), MDA and IL-4 levels but increased SOD, CAT and IFN-γ levels and IFN-γ/IL-4 ratio (193). Onion attenuates bronchial asthma as it relaxes tracheal smooth muscle and causes decreases in neutrophil, eosinophil and lymphocyte counts, inflammatory cytokines (*e.g.* IL-5 and IL-13), eosinophil peroxidase activity and protein exudation in bronchoalveolar lavage fluid. Some constituents also have these protective effects. Flavonoids (*e.g.* quercetin) act as an anti-asthmatic through the reduction of inflammatory indices (*e.g.* NF-κB, PGD2, leukotrienes and granulocyte macrophage-colony stimulating factor (GM-CSF)) and suppression of T helper (Th) 2 cytokine synthesis (*e.g*. IL-4 and IL-13) (194). Treatment with onion extract resulted in increased levels of CAT and SOD activity in rats exposed to nicotine. This increase in antioxidant factors was greater in all tissues than in the vitamin E treated group. Besides, treatment with onion supplementation decreased lipid peroxidation and increased all antioxidant markers in the liver tissue nicotine-treated rats (194). Onion also showed protective effects on carbon monoxide-induced tissue damage in rats by decreased oxidative stress metabolites such as protein carbonyl (PC), MDA, isoprostanes (F2IsoP) levels and increased SOD, CAT, and GPx (195). Also, it was reported that onion extract attenuates the pathological effect of nicotine in lung rats by antioxidative and anti-lipid peroxidative mechanisms (196). Onion also could be effective as a therapeutic agent in allergic rhinitis allergic. In addition, onion and its constituents could be considered as bronchodilatory drugs for the treatment of obstructive pulmonary diseases (197).

It has been suggested that flavonoids (*e.g.* quercetin) present in onion possess anti-asthmatic properties through a reduction in oxidative markers including MDA and inflammatory indices (*e.g*. NF-κB, PGD2, leukotrienes and GM-CSF), elevation in antioxidants such SOD and suppression of Th2 cytokine synthesis (*e.g.* IL-4 and IL-13) (198-202). Quercetin and polyphenols can also inhibit the release of histamine from basophils and mast cells and modulate the process of allergic sensitization (203-206). Moreover, the anti-inflammatory activity of quercetin and its inhibitory effect on muscarinic receptors in bronchial smooth muscle cells prevent bronchoconstriction in asthma (207). In addition to quercetin, kaempferol, thiosulfinates and cepaenes isolated from onion prevent bronchoconstriction by inhibiting cyclooxygenase and lipoxygenase enzymes (133, 208). In mice models of asthma, isoquercitrin or quercetin, reduced eosinophil counts in the bronchoalveolar lavage fluid (BALF), blood and lung parenchyma, neutrophil counts in blood but only isoquercitrin reduced IL-5 levels in lung homogenate (209). In asthma models of mice, quercetin reduced levels of IL-4 and Th2 cytokine, but increased IFN-γ and Th1 cytokine production which indicates the therapeutic effect of quercetin on asthma by improving Th1/Th2 balance (210). Another constituent of the plant, resveratrol, can improve a skewed balance of Th1/Th2 and suppress antigen-specific IgE antibody formation (209-211). Other constituents of onion such as Alkenys also inhibited 5-lipoxygenase, histamine release, leukotriene (LT) B4 and LTC4 biosynthesis from human polymorphonuclear leucocytes, thromboxane B2 (TXB2) biosynthesis by human platelets and allergen (212). The anti-asthmatic and anti-inflammatory effects of onions depend in part on the thiosulfinate moiety.

Topical administration of onion extract on nasal cavity mice model of allergic rhinitis caused a reduction of allergic symptom score, serum levels of total and ovalbumin (OVA)-specific IgE, cytokine levels of nasal mucosa including IL-4, IL-5, IL-10, IL-13, IFN-γ, TNF-α and COX-2 and eosinophilic infiltration. Therefore, topical application of onion extract decreased allergic symptoms by reducing the allergic inflammation and suppressing the Th1 and Th2 cytokine release (30). 

The potent and concentration-dependent relaxant effect of *A. cepa* extract on tracheal smooth muscle compared to the effect of theophylline was also reported due to its β2-adrenergic stimulatory and/or calcium channel blockade possible mechanisms (213). Quercetin also showed a concentration-dependent inhibition of tracheal smooth muscle contractions induced by carbachol and electrical field stimulation (214). Alkenes compounds of onion inhibited platelet activating factor (PAF)-induced bronchial obstruction in guinea pigs (212). It was also shown that Isoquercitrin inhibited tracheal smooth muscle contractile response to OVA and LTD4 (215). The above studies suggest the bronchodilatory effect of onion and its constituents on obstructive pulmonary diseases. 

However, some studies showed an allergic reaction to onion due to the production of IgE and cell-mediated mechanisms against plant lipid transfer proteins (LTPs) (216) which was considered as a cause of asthma induced by the handling of onions. However, despite the wide use of onion in food, few studies reported allergic reactions due to onion ingestion (217).

**Figure 8:**
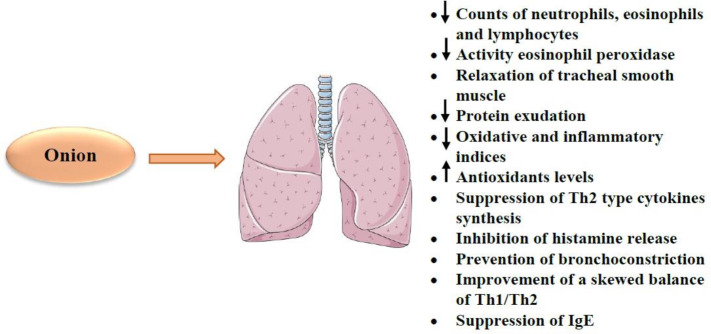
The effects of *A. cepa* (onion) and its constituents on respiratory system. ↓: Decrease; ↑: Increase; Th1: T helper type 1; Th2: T helper type 2; IgE: Immunoglobulin E

The effects of onion and its constituents on the respiratory system were indicated in Table 5. The effects of onion and its constituents on the respiratory system were shown in Figure 8.

Considering the above mentioned, onion has beneficial effects on the respiratory system. For example, this plant attenuates bronchial asthma as it relaxes tracheal smooth muscle and causes the decreases in neutrophil, eosinophil and lymphocyte counts, inflammatory cytokines (*e.g.* IL-5 and IL-13), eosinophil peroxidase activity and protein exudation in bronchoalveolar lavage fluid. Some constituents also have these protective effects. Flavonoids (*e.g.* quercetin) act as an anti-asthmatic through the reduction of inflammatory indices (*e.g*. NF-κB and PGD2, leukotrienes and GM-CSF) and suppression of Th2 cytokine synthesis (*e.g*. IL-4 and IL-13). Quercetin and polyphenols inhibit the release of histamine and modulate the process of allergic sensitization. Quercetin, kaempferol, thiosulfinates and cepaenes can prevent bronchoconstriction. Resveratrol improves a skewed balance of Th1/Th2 and suppresses antigen-specific IgE antibody formation.

*Urogenital effects*

Onion increases libido and strengthens reproductive organs against sexual impotence (5). An *in-vivo* study was designed to evaluate the androgenic effects of onion on spermatogenesis. It was observed that treatment with fresh onion juice significantly increased sperm motility and sperm viability as well as decreased the percentage of sperm head abnormalities (44). The protective mechanism of onion juice might be due to antioxidant compounds (*e.g.* vitamin C) against oxidative stress which has been demonstrated as one factor that affects fertility (23). Several studies have shown that quercetin found in onion has anti-protozoal effects and causes reduction of prostate weight together resulting in the decrease of prostate cancer risk. Quercetin administration also improves prostatitis and prostatodynia (218-220). In an animal study of polycystic ovary syndrome (PCOS), oral treatment with onion for 60 consecutive days increased levels of total antioxidant capacity (TAC), SOD and CAT and reduced MDA (221). Consumption of onion also leads to modulation of the apoptotic process in the ovary by increasing the level of antioxidants (222). Orally treatment of red onion scales (75, 150, and 300 mg/kg/day for 30 days) in Atypical prostatic hyperplasia (APH)-induced prostatic rats ameliorated hyperplasia and inflammation as well as increased apoptosis and suppressed expressions of the inflammatory cytokines such as tissue IL-6, IL-8, TNF-α and insulin-like growth factor 1 (IGF-1) (219). The onion extract is known to elevate testosterone levels which the mechanism of this increase is increasing production of luteinizing hormones, elimination of free radicals by strengthening the antioxidant defense system in the testicular tissue and altering the activity of the adenosine 50-monophosphate as well as activating protein kinase enzyme (223).

**Figure 9:**
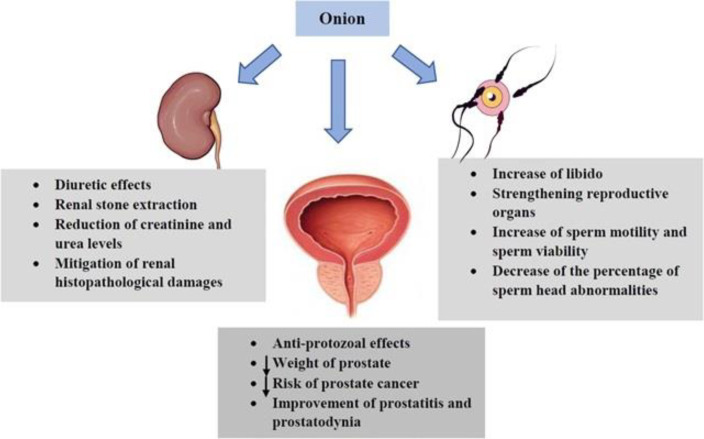
The effects of *A. cepa* (onion) and its constituents on urogenital system. ↓: Decrease

It has been reported that onion possesses a diuretic effect. In addition, the syrup of the plant is useful in extracting renal stones (5). A recent attempt was made to investigate the nephroprotective activity of onion in gentamicin-induced nephrotoxicity. The authors indicated that onion treatment dose-dependently reduced the elevated serum creatinine (Cr) and mitigated renal histopathological damages (*e.g.* dilated tubules and degeneration of epithelial lining) (24). In another study, onion significantly decreased the plasma level of urea in alloxan-diabetic rats (123). The effects of onion and its constituents on the respiratory and urogenital system were reported in Table 5. The effects of onion and its constituents on the urogenital system were demonstrated in Figure 9.

As indicated, onion has beneficial effects on the urogenital system. This plant causes increases in libido, sperm motility and sperm viability, as well as the decrease of the sperm head abnormalities and improvement of sexual impotencegenesis. In addition, quercetin can improve prostatitis and prostatodynia and reduce prostate cancer risk. Onion has a diuretic property and results in extracting renal stones. This plant reduces the elevated Cr and urea levels and attenuates renal histopathological damages.

**Table 1:** Anti-cancer effects of *A. cepa* (onion) and its constituents

**Ref.**	**Effect**	**Extract/Constituent**	**Experimental model**	
(67)	Chemo-preventive effects	Diallyl sulfide, disulfide, dipropyl sulfide and dipropyl disulfide	Exper.	Anti-cancer effects
(212)	Inhibited all stages of skin tumorigenesis	Onion oil		
(30)	Inhibited metastatic potential and growth of melanoma	Quercetin, apigenin, epigallocathechin-3-gallate and resveratrol		
(213, 221)	Inhibition of growth and proliferation of cancer cell lines	Quercetin		
(64)	Increased intestinal absorption, decreased first-pass metabolism of tamoxifen	Quercetin		
(63)	Prevention of radio-resistance following radiotherapy	Phenolic compounds		
(65)	Increased bioavailability of anti-cancer drugs	Flavonoids		
(214)	Inhibited the formation of DNA adducts and proliferation of several tumor cell lines, increased glutathione S-transferase activity	Organosulfur compounds		
(215)	Increased glutathione S-transferase activity, decreased the number of aberrant crypt foci (precursors of colon cancers)	Organosulfur compounds		
(112, 113)	Inhibited the proliferation of several cancer cell lines	Saponins		
(98)	Decreased the brain cancer risk	Onion		
(221)	Decreased the lung cancer risk	Flavonoids		

Exper.: experimental; Clin.: clinical.

**Table 2:** Anti-diabetic and anti-platlet effects of *A. cepa* (onion) and its constituents

**Ref.**	**Effect**	**Extract/** **Constituent**	**Experimental model**	
(114)	Decreased blood glucose levels, increased serum insulin levels	Onion oil	Exper.	Anti-diabetic effects
(116)	Decreased hyperglycemia	Onion powder		
(123)	Decreased serum glucose levels	Onion juice		
(124)	Decrease of hyperglycemia	Onion solution		
(125)	Normalized blood glucose levels, glucose 6 phosphatase and liver hexokinase activities	S-methyl cysteine sulfoxide		
(127)	Increased production and secretion of insulin, decrease in dietary glucose absorption	S-allyl cysteine sulfoxide		
(5)	Decreased blood glucose levels	allyl propyl disulfide and chromium		
(128, 129)	Inhibited the release of D-glucose from oligosaccharides and disaccharides, delayed glucose absorption from small intestine	Quercetin		
(44, 131)	Decreased serum glucose levels, increased serum insulin levels, protection of pancreatic islets	Quercetin		
(118)	Decreased blood glucose levels	Onion	Clin.	
(125)	Control of post-prandial glucose levels	Onion juice		
(5)	Fibrinolytic effects, increased the coagulation time	Onion oil	Exper.	Anti-platlete effects
(141)	Inhibited platelet aggregation	Aqueous extract of raw onion		
(142)	Inhibited platelet aggregation	Methanol extract of onion		
(133, 134, 136)	Inhibited platelet aggregation and thromboxane B_2_ synthesis	Organosulfur components and quercetin		
(132)	Inhibited platelet aggregation, suppressed the formation of all oxygenase products	Onion oil	Clin.	

Exper.: experimental; Clin.: clinical

**Table 3:** The effects of *A. cepa* (onion) and its constituents on bone and cardiovascular system

**Ref.**	**Effect**	**Extract/** **Constituent**	**Experimental model**	
(144-146)	Inhibited bone resorption, increased total bone mineral content	Onion	Exper.	Bone effects
(149)	Inhibited osteoclastogenesis	Water solution of onion crude powder		
(152)	Inhibited osteoclastogenesis and osteoclast formation	Quercetin		
(5)	Inhibited osteoclast activity	Propenyl-cysteine sulfoxide		
(37, 153)	Decreased bone loss, inhibited osteoclast differentiation	Phytoestrogens		
(143)	Slowing down bone resorption, increased osteoblastic activity	Rutin		
(5)	Inhibited osteoclasts activity	Gamma-L-glutamyl-trans-S-1-propenyl-L-cysteine sulfoxide	Clin.	
(46)	Decreased hip fracture	Vitamin K		
(156)	Decreased systolic blood pressure	Aqueous extract of onion	Exper.	Cardiovascular system
(160)	Vasodilation	Raw green-leaf extract of onion		
(165)	Decreased high blood pressure and cardiac hypertrophy, increased nitric oxide availability	Quercetin		
(162, 163)	Decreased myocardial infarction incidence, protected the initiation and progression of atherosclerosis	Flavonoids	Clin.	
(164)	Decreased coronary heart disease incidence	Isoflavones		

Exper.: experimental; Clin.: clinical.

**Table 4:** The effects of *A. cepa* (onion) and its constituents on gastrointestinal and nervous systems

**Ref.**	**Effect**	**Extract/** **Constituent**	**Experimental model**	
(174)	Decreased food transit time in gastrointestinal system	Onion	Exper.	Gastrointestinal system
(123)	Decreased aspartate aminotransferase, alanine aminotransferase and lactate dehydrogenase activities	Onion juice		
(40, 175)	Stimulated growth of useful microorganisms in the colon	Fructans		
(176)	Anti-spasmodic and anti-diarrheal effects	Flavonoids		
(177)	Anti-ulcer effect	Quercetin		
(178)	Increased the amount of gastric mucus, decrease of gastric lesions	Quercetin		
(182)	Improved memory and behavioral deficits, motor incoordination and short-term memory	Onion	Exper.	Nervous system
(5, 182)	Decreased cerebral infarct size and improved motor incoordination and short-term memory	Methanol extract of onion		

Exper.: experimental; Clin.: clinical.

**Table 5:** The effects of *A. cepa* (onion) and its constituents on respiratory and urogenital systems

**Ref.**	**Effect**	**Extract/** **Constituent**	**Experimental model**	
(189)	Anti-asthma effects	Thiosulfinates and cepaenes	Exper.	Respiratory system
(190)	Decreased bronchial obstruction	Alcoholic extract of onion		
(44)	Decreased cellular infiltration (eosinophil and lymphocyte) and lung inflammation	Aqueous extract of onion		
(191)	Relaxation of tracheal smooth muscle	Methanolic extract of onion		
(193)	decreased immunological markers interleukin-4 and immunoglobulin E, increased interferon-γ	Onion		
(199)	Inhibited interleukin-4 production	Flavonoids		
(203)	Prevented mast cell and basophil degranulation	Quercetin		
(203)	Prevented histamine secretion	Vitamin C		
(206)	Decreased airway hyperreactivity	Quercetin		
(207)	Anti-allergic effects	Kaempferol		
(208)	Improved T helper 1/T helper 2 balance, suppression immunoglobulin E formation	Resveratrol		
(200)	Anti-asthma effects	Flavonoids	Clin.	
(203)	Inhibited histamine release	Quercetin		
(203)	Decreased nasal secretions and edema	Vitamin C		
(23, 44)	Improved testes weight, increased serum testosterone levels, sperm count, viability and motility	Juice of onion bulbs	Exper.	Urogenital system
(210)	Decreased cellular proliferation, inflammation and apoptosis in atypical prostatic hyperplasia	Methanolic extract		
(24)	Decreased serum creatinine level, protected kidneys from oxidative stress	Onion		
(123)	Decreased serum urea and creatinine levels	Onion juice		
(5)	Increased libido and strengthening reproductive organs against sexual impotence	Onion	Clin.	
(216)	Improved non-bacterial chronic prostatitis and prostodynia	Quercetin		
(5)	Diuretic effect	Onion		
(5)	Extraction of renal stones	Syrup of onion		

Exper.; experimental, Clin.: clinical.

## Conclusion

This review showed a wide spectrum effects of *A. cepa* or onion and its constituents on different disorders both in experimental and clinical studies. Regarding the anti-cancer effects of the plant and its constituents, chemo-preventive and inhibitory effects of the plant on all stages of skin tumorigenesis, inhibitory potential of quercetin on metastatic and growth of melanoma, growth and proliferation of cancer cell lines and its effects of increased intestinal absorption and decreased the first-pass metabolism of tamoxifen were shown in experimental studies. In other experimental studies, the inhibitory effects of the proliferation of several cancer cell lines, the formation of DNA adducts, an increase of glutathione S-transferase activity, a decrease of the number of aberrant crypt foci (precursors of colon cancers) and prevention of radio-resistance following radiotherapy were also indicated for other constituents of the plant. In addition, the reduction effects of onion on the brain cancer risk and decrease of the lung cancer risk for its flavonoids were reported in clinical studies.

The reduction effects of onion and its components, mainly quercetin, on blood glucose levels and dietary glucose absorption and increase of serum insulin levels, as well as protection of pancreatic islets, were demonstrated in experimental studies. In clinical trials, also the reduction effects of onion on blood glucose levels were seen.

The inhibitory effects of onion and its constituents, such as organosulfur components and quercetin on platelet aggregation and TXB2 synthesis, were indicated in experimental studies. The inhibitory effects of the plant on platelet aggregation and suppression of the formation of all oxygenase products were also documented in clinical studies.

In experimental studies, the inhibitory effects of the plant and its components such as quercetin and rutin on bone resorption, osteoclastogenesis as well as osteoclast formation and activity, increase of total bone mineral content and osteoblastic activity, a decrease of bone loss were reported. In clinical studies, the inhibitory effects of onion on osteoclasts activity and decreased hip fracture for vitamin K were demonstrated.

In the cardiovascular system, the reduction effects of onion on high blood pressure and cardiac hypertrophy, vasodilation and increased nitric oxide availability for quercetin in experimental research were seen. The flavonoids of the plant also showed decrease incidence in myocardial infarction and coronary heart disease and protection of the initiation and progression of atherosclerosis in clinical trials.

On gastrointestinal system, decreases in food transit time in the gastrointestinal system as well as aspartate aminotransferase, alanine aminotransferase and lactate dehydrogenase activities were shown for onion and increased growth of useful microorganisms in the colon, anti-spasmodic, anti-diarrheal and anti-ulcer effects, increased amount of gastric mucus and decreased gastric lesions were shown for the plant ingredients mainly quercetin.

Onion also improved the memory and behavioral deficits, motor incoordination and short-term memory and decreased cerebral infarct size.

The anti-asthmatic effects and relaxation of tracheal smooth muscle, a decrease of cellular infiltration (*e.g.* eosinophil and lymphocyte) and lung inflammation as well as reduction of immunological markers such as IL-4 and IgE and increase of IFN-γ were reported for onion in experimental studies. The constituents of the plant also showed inhibitory effects on IL-4 production, IgE formation, mast cell and basophil degranulation, histamine secretion and airway hyperreactivity as well as improvement of Th1/Th2 balance and anti-allergic effects. In clinical trials, the plant constituents showed anti-asthmatic activities, inhibition of histamine release and decreases in nasal secretions and edema.

In the urogenital system, onion also showed improvement of testes weight, increases in serum testosterone levels, sperm count, viability and motility, decreases in cellular proliferation, inflammation and apoptosis in atypical prostatic hyperplasia, reduction of serum creatinine levels and protection of kidneys from oxidative stress in experimental studies. In clinical studies, onion also showed increased libido and strengthening reproductive organs against sexual impotence, improved non-bacterial chronic prostatitis and prostodynia, diuretic effects and extraction of renal stones.

Therefore, a wide range of pharmacological effects was reported for onion in the published studies, mainly in experimental studies. However, more clinical trials are needed regarding the effects of the plant and its constituents before they could be used in clinical practice.
